# An assessment of routine primary care health information system data quality in Sofala Province, Mozambique

**DOI:** 10.1186/1478-7954-9-12

**Published:** 2011-05-13

**Authors:** Sarah Gimbel, Mark Micek, Barrot Lambdin, Joseph Lara, Marina Karagianis, Fatima Cuembelo, Stephen S Gloyd, James Pfeiffer, Kenneth Sherr

**Affiliations:** 1 Department of Global Health, School of Public Health and Community Medicine, University of Washington. Seattle, WA 98195, USA; 2 Health Alliance International, 4534 11th Ave NE, Seattle, WA 98105, USA; 3 Health Alliance International-Beira, Rua Aires de Ornela Nr. 458 R/C, Beira, Mozambique; 4 Provincial Department of Health-Sofala Province, Rua Mayor Serpa Pinto 294, 4 Andar, Sector Reparticao de Saude da Communidade, Beira, Mozambique; 5 Eduardo Mondlane University, Community Health Department, Maputo, Mozambique

## Abstract

**Background:**

Primary health care is recognized as a main driver of equitable health service delivery. For it to function optimally, routine health information systems (HIS) are necessary to ensure adequate provision of health care and the development of appropriate health policies. Concerns about the quality of routine administrative data have undermined their use in resource-limited settings. This evaluation was designed to describe the availability, reliability, and validity of a sample of primary health care HIS data from nine health facilities across three districts in Sofala Province, Mozambique. HIS data were also compared with results from large community-based surveys.

**Methodology:**

We used a methodology similar to the Global Fund to Fight AIDS, Tuberculosis and Malaria data verification bottom-up audit to assess primary health care HIS data availability and reliability. The quality of HIS data was validated by comparing three key indicators (antenatal care, institutional birth, and third diptheria, pertussis, and tetanus [DPT] immunization) with population-level surveys over time.

**Results and discussion:**

The data concordance from facility clinical registries to monthly facility reports on five key indicators--the number of first antenatal care visits, institutional births, third DPT immunization, HIV testing, and outpatient consults--was good (80%). When two sites were excluded from the analysis, the concordance was markedly better (92%). Of monthly facility reports for immunization and maternity services, 98% were available in paper form at district health departments and 98% of immunization and maternity services monthly facility reports matched the Ministry of Health electronic database. Population-level health survey and HIS data were strongly correlated (R = 0.73), for institutional birth, first antenatal care visit, and third DPT immunization.

**Conclusions:**

Our results suggest that in this setting, HIS data are both reliable and consistent, supporting their use in primary health care program monitoring and evaluation. Simple, rapid tools can be used to evaluate routine data and facilitate the rapid identification of problem areas.

## Introduction

The 2008 World Health Report issued a call for a renewed focus on primary health care (PHC). As set forth in the 1978 Alma Ata Declaration, the ultimate goal of PHC is better health for all through a reduction in exclusion and social disparities in health, the organization of health services around people's health needs and expectations, the integration of health into all sectors through effective policy planning, and the pursuit of collaborative models of policy dialogue through increasing stakeholder participation.[1] A key systems building block of PHC is an intact health information system (HIS), which generates information to enable decision-makers at all levels of the health system to identify problems and needs, make evidence-based decisions on health policy, and allocate scarce resources optimally.[2] Effective monitoring and supervision of health care programs depend on complete, accurate, and timely flow of data between primary health care facilities, hospitals, and a central information hub. Ultimately, effective use of information has been identified as a key element in the success of large-scale efforts that have achieved major health improvements.[3]

There are six main types of recognized surveillance data, including census, vital events, disease-specific registries, administrative data, household surveys, and national health accounts. Within the framework of PHC, administrative data provide the best opportunity for public health systems to capture facility-level information to guide program planning and management. Administrative data are better suited to PHC than other forms of surveillance in this regard because they are universal in scope, inexpensive, and data are immediately available at each level of the health system. This allows clinic-level managers, as well as provincial and national managers, to use the information in either aggregated or nonaggregated formats to guide decision-making and program implementation.

Concerns about the quality of routine administrative data in resource-limited settings such as Mozambique have undermined their use [4,5]. Earlier studies and reports have found that the data received by health managers from health facilities in resource-limited settings are chronically incomplete, inaccurate, and untimely [6,7]. One study, carried out in 2004 in selected districts in southern Mozambique to assess the quality of routine malaria data, found primary data to be of poor quality and that the multiplicity of reporting channels of information (4) contributed to a duplication of efforts and ultimately to low validity, incorrectness, and incompleteness of data. In addition, there are typically limited human resources available to analyze and translate data into useful information for health managers. Acting on useful information can also be challenging when program staff have different data needs from surveillance experts. Surveillance systems are often donor-driven and developed to monitor vertically-funded priority initiatives, further undermining and directing resources away from the PHC system.[8,9] As the donor community recommits to the importance of PHC, the focus on strengthening routine HIS is crucial to ensure that information collection is funded systematically rather than categorically. The ultimate goal of a national HIS is the coordination of government and donor information needs and flows through one national HIS to decrease system fragmentation, minimize duplicative and burdensome reporting, and improve data quality and use.[2] In this context, our aim was to evaluate the strengths and weaknesses of HIS in three districts in central Mozambique, by evaluating the quality of HIS data prior to the initiation of a seven-year joint PHC strengthening project of the Mozambican Ministry of Health (MOH), the University of Eduardo Mondlane, and the University of Washington (UW)/Health Alliance International (HAI), funded by the Doris Duke Charitable Foundation/African Health Initiative. The intention of this paper was not to assess other aspects of HIS data use, such as determinants of data quality or use in the aforementioned three districts.

## Methods

### Ethics

This study was approved by the Institutional Review Board (IRB) of the Mozambique National Institute of Health. Co-investigators also discussed the study with the IRB at the UW, which determined that the UW IRB review was not necessary because the primary intent of the project was for program evaluation purposes, and therefore not considered human subjects research under United States federal regulations.

### Study setting and sites

Sofala Province, with a population of approximately 1.6 million, is similar to Mozambique as a whole in terms of high disease burden (23% HIV prevalence) and limited availability of health workers (three medical doctors and 21 nurses per 100,000 inhabitants) [10]. Sofala Province is traversed by major highways connecting its provincial capital city, Beira, with Manica Province and Zimbabwe to the west and smaller roads connecting to Zambezia Province in the north and to Inhambane Province to the south. Sofala was heavily affected during the 20-year civil war, which decimated the health infrastructure. HAI, a nongovernmental organization (NGO), is a main external support organization for the health sector in the Province. HAI has over 20 years of experience working with the Mozambican MOH and began working in Sofala Province in 1995.

In Sofala Province, there are a total of 137 primary-level health facilities (12 urban health centers, 10 rural health centers type I, 89 rural health centers type II, and 26 health posts), four secondary-level health facilities (rural hospitals), and one quaternary health facility (central hospital). There is only one notable private clinic in Sofala Province, located in the provincial capital (Beira), which provides a nonconsequential level of formal health services (estimated to be less than 1% of facility-assisted births and outpatient consults in the entire province, as reported by the HIS).

For this study, we assessed HIS data quality in nine government-run health facilities within three districts (Beira City, Dondo, and Caia) of Sofala Province in central Mozambique. The researchers employed a purposive sampling approach, selecting three focus districts based on their representativeness of the three main types of districts in Sofala Province--urban, peri-urban, and rural--and because of their accessibility to the researchers. In Beira City, three large urban health centers were selected as demonstrative of sites where most people receive PHC services. The largest hospital in the city (Beira Central Hospital) was excluded because it does not provide core PHC services, such as immunization and antenatal care services. The health facilities chosen from the other two districts were selected to represent a broad range of PHC facilities in those locales, and included from each district: 1) a large district hospital/health center, 2) a medium-sized health center outside the district capital, and 3) a small rural health center/health post.

Mozambique's National Health Service (NHS) is the key organizational unit through which PHC services are managed, coordinated, and brought to scale. Since its inception in 1975, the NHS has been designed to provide integrated primary health care services through a widespread network of health facilities distributed across the country. Chronic resource shortages, vertical funding from donors, and management challenges all limit service coverage and quality. The Mozambique health system is currently undergoing a decentralization process, which will further devolve important management and planning tasks from the provincial to the district level. Unfortunately, most district health directorates remain under-resourced with limited technical, managerial, and workforce capacity to assume these devolved responsibilities. District management is further hampered by a combination of relatively weak data collection systems and low district capacity to analyze data for district-level decision-making and planning.

**Figure 1:**
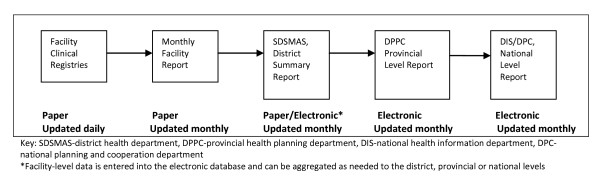
**Mozambique health information system flow map**.

The Mozambique HIS is similar to the data systems of other countries in the region, incorporating both paper and electronic elements, depending on the health system level. Patient-level information is collected daily in facility paper-based clinical registries, typically by nurses within health facilities (Figure 1). On a monthly basis, key indicators are culled from the facility clinical registries and aggregated into monthly facility reports, which are sent on to the district level. In most cases, the district planning and statistics department enters the monthly facility reports into the MOH electronic database, known as the "Basic Module," and the electronic files are sent to the provincial level, where they are aggregated and forwarded to the national level via email or on flash drives.

### Data collection and statistics

For the purposes of this activity, we used a methodology similar to the Global Fund to Fight AIDS, Tuberculosis and Malaria (GFATM) on-site data verification bottom-up audit tool [11] to assess the primary health care HIS, which included the following components (see Table 1):

**Table 1:** Population and patient data sources of information

	Indicator	Source	Reference source	Time period	Analysis
**Availability**	Presence/absence of monthly maternity and immunization program reports on file	Monthly facility reports	Not applicable	Nov. 1, 2007-Oct. 31, 2008	Presence/absence of monthly reports at health facility and district health departments

**Reliability**	First ANC, institutional birth, DPT3, HIV testing, and outpatient consults	Facility clinical registries, monthly facility reports	Facility clinical reports	July 1, 2008-Dec. 31, 2008	Percent matching months between facility clinical registries and monthly facility reports, percent difference for non-identical months
	
	Immunization and maternity reports*	Monthly facility reports, MOH electronic database	Monthly facility reports	Nov. 1, 2007-Oct. 31, 2008	Percent matching months between monthly district reports and MOH electronic database

**Validity**	First ANC, institutional birth, DPT3	Provincial annual health report (data from MOH electronic database)	DHS, MICS	1995-2008	Percent of provincial annual health report figures falling within the 95% confidence intervals of DHS/MICS survey estimates, correlation coefficient from weighted linear regression
					
		DHS		1997, 2003	
					
		MICS		2008	

*Indicators in immunization report include target group, tetanus first dose, tetanus second to fifth dose, total tetanus doses, coverage rate/goal, doses used, and wastage rate. Indicators in maternity reports include institutional birth, live births, low-weight births, stillbirths, stillbirths with heartbeat upon hospital admission, discharges, total inpatient days.

(Key for abbreviations--ANC: antenatal care, DPT3: third diptheria, pertussis, and tetanus immunization, MOH: Ministry of Health, DHS: Demographic Health Survey, MICS: Multiple Indicator Cluster Survey)

1. Verification of the *availability *of monthly facility reports at the health facility and district health departments

2. Evaluation of the *reliability *(concordance) of monthly statistics obtained from facility clinical registries, monthly facility reports, and the MOH electronic database

3. Examination of the *validity *of the HIS data by comparison with population-level surveys over time

The GFATM data verification tool includes the first and second components but not the third, which was added by the researchers as a means to assess the validity of the routine HIS data compared to population-level surveys that are powered for provincial-level analysis.

Another important difference between the GFATM data verification tool and our methodology was the selection of indicators representative of PHC services, rather than indicators from the GFATM focus diseases (HIV, tuberculosis, and malaria). However, we used the GFATM data verification rating system to classify the concordance of the reliability indicators according to the following scale:

A - less than 10% error margin

B1 - between 10% to 20% error margin

B2 - above 20% error margin

C - no systems in place

All data were collected by provincial level health managers and NGO counterparts based at the MOH's Beira Operations Research Center (BORC). This applied research center is in no way involved with the management of the health system, nor in the delivery of health services, and thus its employees are uniquely poised to evaluate how the health system operates. In addition, the training provided to the data collection staff also mitigated potential bias in data collection. It should be noted that district and facility staff did help in providing the data registries and monthly reports but were not involved in collecting data.

### Data availability

To assess the availability of monthly health facility and district reports for immunization and maternity services, we noted the monthly presence or absence of these two reports over a 12-month period (November 1, 2007 through October 31, 2008) at both health facilities and district health departments (Table 1). Ideally, paper copies of the monthly facility reports should be organized chronologically in binders at the health facility, and original reports should be sent to the district health department offices, where paper copies are kept and filed after they are entered into the MOH electronic database. Provincial-level health managers and NGO counterparts from the BORC worked together to collect this information over a one-week period.

### Data reliability

To assess data reliability, we recorded monthly figures from facility clinical registries and monthly facility reports for five key indicators (first antenatal care [ANC1], institutional birth, DPT3, HIV testing, and outpatient consults) for the six-month period from June through December 2008, which was deemed adequate to gain a sufficient picture of data reliability over time (Table 1). We then calculated the proportion of facility-months in which the data were not identical and calculated the percentage difference for those months where data were not identical.

We also compared recorded monthly figures from aggregated district reports and the MOH electronic database (obtained at the provincial level) for immunization and maternity services for a 12-month period from November 2007 to October 2008. Seven indicators from the immunization report were included: target group, first tetanus dose, second to fifth tetanus dose, total tetanus doses, coverage rate/goal, doses administered, and wastage rate. Eight indicators from the maternity reports were included: institutional births, live births, low-weight births, stillbirths, stillbirths with heartbeat upon hospital admission, maternal deaths, discharges, and total inpatient days. We calculated the proportion of district months where data were not identical between the district reports and the MOH electronic database and the percentage difference for those months when data were not identical. Data collection for assessing data reliability was carried out by MOH provincial level health managers and NGO counterparts over a period of two weeks.

### Data validity

To assess data validity, we compared statistics supplied from the provincial health department's annual reports (which use data from the MOH electronic database) with those obtained from the 1997 and 2003 Mozambique Demographic Health Surveys (DHS) and the 2008 Multiple Indicator Cluster Survey (MICS). The DHS and MICS surveys were national-level community-based surveys, carried out in Mozambique following standardized and internationally-recognized methodology (Table 1).[12,13]

For this analysis, we focused on three provincial-level indicators that were representative of PHC and obtainable from all three aforementioned sources: ANC coverage, institutional birth coverage, and DPT3 coverage. For the surveys, ANC coverage was defined as births that received ANC from a trained health professional during pregnancy, institutional birth coverage was defined as births that occurred in a health facility, and DPT3 coverage as the proportion of children 12 to 23 months old who had received three DPT immunizations (from documentation on immunization cards or mothers' reports) at the time of the survey. Due to data constraints for the variable indicating site of delivery in the MICS dataset, we estimated institutional birth coverage from this dataset using a different question ascertaining whether births were attended by a doctor, nurse, or midwife. In the DHS, the concordance between these two items was very good (33.9% vs. 34.0% in the 1997 DHS and 57.4% vs. 56.1% in the 2003 DHS, for births in a health facility versus births attended by a doctor, nurse, or midwife, respectively), justifying its use in the 2007 MICS dataset.

For ANC and institutional birth coverage, we harmonized the DHS and MICS by including responses from women who had given birth in the previous two years before the survey (1995-1996 for the 1997 DHS, 2001-2002 for the 2003 DHS, and 2006-2007 for the 2008 MICS). We estimated DPT3 coverage up to two (1997 DHS) and four (2003 DHS and 2008 MICS) years prior to the survey. We utilized the responses recorded for children aged 12-23 months, 24-35 months, 36-47 months, and 48-59 months to estimate coverage for 1 year, 2 years, 3 years, and 4 years prior to the survey, respectively.

MOH statistics were collected from the Sofala provincial health department annual reports, which were derived from the MOH electronic database for the numerators, and census data for the denominators. DHS and MICS metrics were obtained from the original DHS and MICS datasets from http://www.measuredhs.com and the Mozambican National Institute of Statistics, respectively. For all DHS and MICS analyses, we used only data from Sofala Province to obtain provincial-level estimates.

Secular trends of HIS data derived from the MOH electronic database and the community level surveys (DHS and MICS) with 95% confidence intervals were plotted. Note that HIS data do not have calculable confidence intervals, as they are not estimates derived from sampling but rather represent the entirety of provincial-level data. To evaluate the degree of correlation between the two data sources, we first determined the proportion of HIS annual estimates that were within the 95% confidence intervals of the corresponding DHS/MICS estimate. Next, we determined the degree of correlation between the annual HIS and corresponding DHS/MICS figures, using a weighted linear regression model with the survey estimate as the dependent variable and the HIS data as the independent variable. The weights were inversely proportional to the variance of the survey estimates. The correlation coefficient was determined by taking the square root of the R-squared estimate. For ANC coverage and institutional birth delivery, we compared the 1995-1996, 2001-2002, and 2006-2007 survey estimates to the mean of the administrative data over these two years, while the yearly DPT3 estimates were compared to the administrative estimate for the corresponding year.

All simple descriptive statistics were carried out using Stata version 11.1 (College Station, Texas, USA).

## Results and discussion

### Data availability

Over the 12-month study period included in analysis of data availability (November 1, 2007 through October 31, 2008), 97% (105/108) of immunization and 99% (107/108) of maternity monthly facility reports were properly on file at the health facility level. At the district health departments, 99% (35/36) of the monthly facility reports for immunization and 100% (36/36) of the maternity reports were properly filed and available. In some cases, health facilities did not make copies of the report to keep on file before sending it on to the district level.

### Data reliability

Over six months (June through December 2008), five indicators were assessed across nine health facilities for data reliability. Three of the sites did not provide HIV testing services, and one site did not have access to the outpatient consult registry at the time of data collection. Therefore, there was no data collection for those sites for those indicators. Exactly 80% (196/246) of data from facility clinical registries and monthly facility reports matched exactly on the five key indicators (Table 2).

**Table 2:** Concordance between health facility clinical registries and monthly facility reports for five key indicators and GFATM data verification ratings

		Number of months in which figures from facility clinical registers match monthly facility reports, for six-month period (June 1, 2008 to Dec 31, 2008), by indicator						
		
		First ANC	Institutional birth	DPT3	HIV testing	Outpatient consults	TOTAL	**GFATM rating grade**
				
District	Health facility type	N (%)	N (%)	N (%)	N (%)	N (%)		
1	Urban	1/6 (17)	2/6 (33)	4/6 (67)	0/6 (0)	4/6 (67)	11/30 (37)	B2
	Peri-urban	5/6 (83)	6/6 (100)	6/6 (100)	NA	6/6 (100)	23/24 (96)	A
	Rural	6/6 (100)	6/6 (100)	6/6 (100)	NA	6/6 (100)	24/24 (100)	A

2	Urban	2/6 (33)	1/6 (17)	2/6 (33)	3/6 (50)	6/6 (100)	14/30 (47)	B2
	Peri-urban	6/6 (100)	1/6 (17)	6/6 (100)	6/6 (100)	NA	19/24 (79)	B2
	Rural	6/6 (100)	4/6 (67)	6/6 (100)	NA	6/6 (100)	22/24 (92)	A

3	Urban	6/6 (100)	6/6 (100)	5/6 (83)	6/6 (100)	6/6 (100)	29/30 (97)	A
	Urban	4/6 (67)	6/6 (100)	5/6 (83)	6/6 (100)	4/6 (67)	25/30 (83)	B1
	Urban	6/6 (100)	6/6 (100)	6/6 (100)	5/6 (83)	6/6 (100)	29/30 (97)	A

TOTAL		42/54 (77)	38/54 (70)	46/54 (85)	26/36 (72)	44/48 (92)	196/246 (80)	B1

NA = not applicable because the service was either not provided at the site (HIV) or not available during the data collection period (outpatient)

(Key for abbreviations--ANC: antenatal care, DPT3: third diptheria, pertussis, and tetanus immunization)

GFATM rating scale--A: less than 10% error margin, B1: between 10% to 20% error margin, B2: above 20% error margin, C: no systems in place.

When two sites, both urban facilities, were excluded from the analysis, the concordance was markedly better (92%) and the GFATM data verification rating jumped from borderline B1/B2 to A (Table 2). Of the 50 monthly figures which did not match exactly, the median percentage difference was 4% (range <.01%-59%) and the majority (86%) differed by 10% or less. Most of the monthly disagreements (35/50, 70%) were from the two larger-district urban health facilities, highlighting that the majority of problems were confined to a few sites. Among the various indicators, the weakest reliability was noted in institutional birth and ANC registration (28/50, 56%) (Table 3).

**Table 3:** Distribution of discordant monthly figures from health facility clinical registries versus monthly facility reports

District	Health facility type	First ANC	Institutional birth	DPT3	HIV testing	Outpatient consults	TOTAL
1	Urban	5	4	2	5	2	18
	Peri-urban	1	0	0	0	0	1
	Rural	0	0	0	0	1	1

2	Urban	4	5	4	3	1	17
	Peri-urban	0	5	0	0	0	5
	Rural	0	2	0	0	0	2

3	Urban	0	0	1	0	0	1
	Urban	2	0	1	0	1	4
	Urban	0	0	0	1	0	1

TOTAL		12	16	8	9	5	50

(Key for Abbreviations--ANC: antenatal care, DPT3: third diptheria, pertussis, and tetanus immunization)

In addition, 96% (242/252) of the monthly aggregated district reports for immunization and 98% (287/288) of monthly aggregated district reports for maternity services matched the information available through the MOH electronic database over the 12-month period.

### Data validity

**Figure 2:**
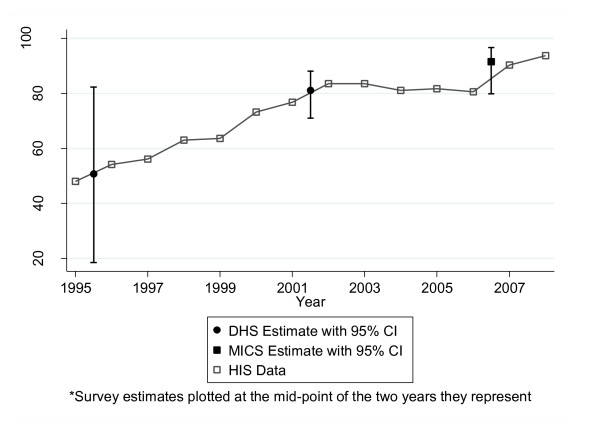
**ANC coverage, Sofala Province, 1995-2007: HIS versus DHS/MICS**.

**Figure 3:**
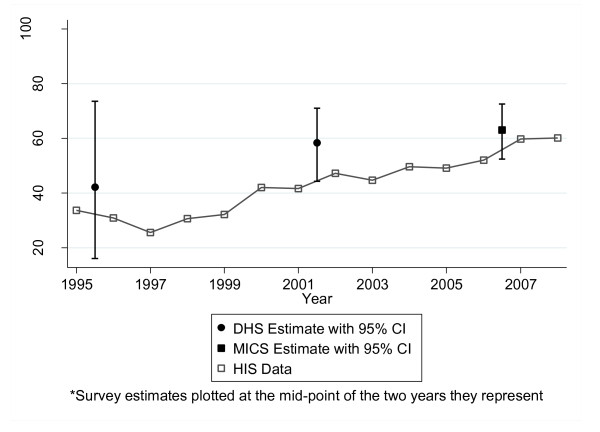
**Institutional birth coverage, Sofala Province, 1995-2007: HIS versus DHS/MICS**.

**Figure 4:**
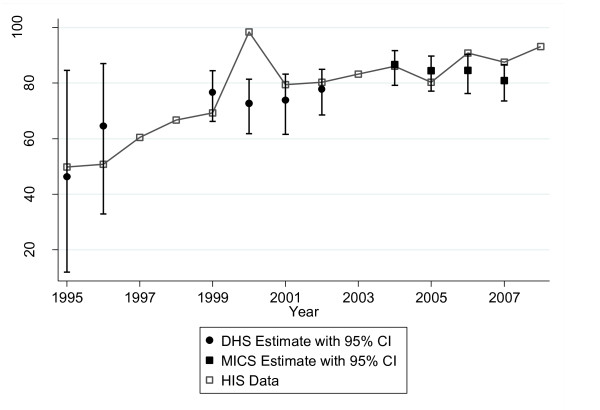
**DPT3 coverage, Sofala Province, 1995-2007: HIS versus DHS/MICS**.

The comparisons of DHS and MICS with MOH electronic database information were similar in terms of absolute results for the three variables considered: ANC coverage, institutional birth coverage, and DPT3 coverage (Figures 2, 3, and 4). Table 4 shows the survey estimates and HIS data for the corresponding time period. Overall, 81% (13/16) of the HIS data were within the 95% confidence interval of the corresponding survey estimate. Data for DPT3 coverage for 2000 (DHS: 72.7 [61.8-81.4] vs. HIS: 98.5), 2006 (DHS: 84.6 [76.3-90.3] vs. HIS: 90.9) and 2007 (DHS: 81.0 [73.5-86.7] vs. HIS: 87.6) were not located within the 95% confidence interval. In comparing the two data sources, the correlation coefficient was 0.73; however, the correlation was 0.88 when the DPT3 data points for both sources from the year 2000 (the point with the most discrepant results in the entire analysis) were excluded.

**Table 4:** DHS/MICS estimates versus HIS data for the corresponding time period

	**DHS/MICS estimate** **(95% CI)**	**HIS data**
	
**Institutional birth coverage**		
1995-1996	42.2 (16.0-73.6)	32.2
2001-2002	58.3 (44.3-71.0)	44.4
2006-2007	63.0 (52.5-72.5)	55.9
**ANC coverage**		
1995-1996	50.7 (18.5-82.3)	51.2
2001-2002	81.1 (71.1-88.2)	80.2
2006-2007	91.5 (79.9-96.7)	85.5
**DPT3 coverage**		
1995	46.3 (11.9-84.6)	49.8
1996	64.5 (32.9-87.1)	50.7
1999	76.6 (66.3-84.5)	69.3
2000	72.7 (61.8-81.4)	98.5
2001	73.9 (61.6-83.3)	79.4
2002	77.9 (68.6-85.0)	80.3
2004	86.6 (79.1-91.7)	86.1
2005	84.4 (77.1-89.8)	80.3
2006	84.6 (76.3-90.3)	90.9
2007	81.0 (73.5-86.7)	87.6

(Key for abbreviations--DHS: demographic health survey, MICS: multiple indicator cluster survey, HIS: Health information system, ANC: antenatal care, DPT3: third diptheria, pertussis, and tetanus immunization)

### Limitations

This study was limited by the relatively small sample of health facilities included, as well as the limited geographic area. In addition, the effect of the disaggregation of data and the use of a small number of sites may have contributed to the very good concordance demonstrated between facility level data and the MOH electronic database. Likewise, aggregation of data at the provincial level may have effectively hidden problematic sites when comparing validity with the findings of population level surveys.

An additional limitation of the study was the use of provincial MOH health managers and NGO counterparts to carry out the data collection. Provincial MOH managers are technically responsible for data quality in the province and thus may have been biased in their collection. Although this may be deemed a limitation by some, the full involvement of MOH managers in this data quality audit was regarded as essential programmatically in order to build local capacity and ownership over the process.

## Conclusions

The results of this evaluation, which used relatively simple, rapid tools, suggest that in this study setting, routine administrative data are adequately available, reliable, and consistent from the health facility to the provincial health department levels. The exercise was also helpful in pointing out discrepancies between clinic registers and monthly facility reports at two district urban health facilities and within the maternity and antenatal care settings. The DHS/MICS and HIS data comparisons confirm that over time at the provincial level, the data are valid and useful for program monitoring, evaluation, and planning. Notably, at the data points where the HIS and DHS/MICS data were the most discrepant (the comparison of DPT3 coverage in 2000), our findings were consistent with previous studies. These studies identified increases in multiple countries in reported coverage of DPT3 based on HIS data in the year following the implementation of a global initiative to expand vaccination coverage, and for which fund distribution was dependent on the coverage rate in the second year (which was 2000 for Mozambique) [5].

Of the many past reports and studies that have found administrative data in developing countries to be of poor quality and unreliable for disease or program surveillance, most have suggested that the root cause of the problem is the lack of significant value assigned by clinic-level health workers to the quality of administrative data collection [6,14]. Indeed, it is widely recognized that health systems rarely use routinely-collected data to improve functioning [15,16]. In order for this to improve, district- and health facility-level managers need expanded skills to allow for full involvement in the process of evaluating their data.

Though this research project was not designed to determine the cause of variation in data reliability, other similar studies carried out in resource-constrained environments have described the importance of human resource levels, management and planning capacity, infrastructure capacity, and the like in determining data quality. The Mozambique health system is faced with significant constraints in these areas, which may explain variation in data reliability across different services.

PHC service-strengthening in Sofala Province should enhance the design, testing, and subsequent use of similar operational research tools to further expand the quality and use of routine data by health managers and policymakers alike. Health managers accustomed to using health systems data and ensuring their quality through bottom-up audits will be invaluable to the ongoing decentralization process and will provide valuable lessons for other areas of Mozambique and possibly sub-Saharan Africa.

## Competing Interests

The authors declare that they have no competing interests.

## Authors' contributions

SG was responsible for the initial conception and design of the data, participated in the data analysis and drafted the original text. MM participated in the data analysis and made significant comments on progressive drafts. BL provided input on the design of the study, provided critical input in the data analysis, and commented on progressive drafts. JL supported data collection and provided input on progressive drafts. MK provided critical input in design of the data and supported the data collection process. FC gave input on the development of the discussion section and gave input on progressive drafts. SSG participated in the initial design of the study and provided input on progressive drafts. JP gave input on the development of the discussion section and helped in revisions of the final draft. KS provided critical input into the initial design of the study and made significant comments on progressive drafts, All authors read and approved the final manuscript.

## References

[B1] Fendall NR Declaration of Alma-Ata Lancet 1978 2 1308 82806 10.1016/s0140-6736(78)92066-4

[B2] World Health Organization Health Metrics Network Framework and Standards for Country Health Information Systems 2008 World Health Organization, Geneva

[B3] Peersman G Rugg D Erkkola T Kiwango E Yang J Are the investments in national HIV monitoring and evaluation systems paying off? J Acquir Immune Defic Syndr 2009 52 Suppl 2 S87 96 19901631 10.1097/QAI.0b013e3181baede7

[B4] Ronveaux O Rickert D Hadler S Groom H Lloyd J Bchir A Birmingham M The immunization data quality audit: verifying the quality and consistency of immunization monitoring systems Bull World Health Organ 2005 83 503 510 16175824 PMC2626295

[B5] Lim SS Stein DB Charrow A Murray CJ Tracking progress towards universal childhood immunisation and the impact of global initiatives: a systematic analysis of three-dose diphtheria, tetanus, and pertussis immunisation coverage Lancet 2008 372 2031 2046 10.1016/S0140-6736(08)61869-3 19070738

[B6] Mate KS Bennett B Mphatswe W Barker P Rollins N Challenges for routine health system data management in a large public programme to prevent mother-to-child HIV transmission in South Africa PLoS One 2009 4 e5483 10.1371/journal.pone.0005483 19434234 PMC2677154

[B7] Garrib A Stoops N McKenzie A Dlamini L Govender T Rohde J Herbst K An evaluation of the District Health Information System in rural South Africa S Afr Med J 2008 98 549 552 18785397

[B8] Pfeiffer J Montoya P Baptista AJ Karagianis M Pugas Mde M Micek M Johnson W Sherr K Gimbel S Baird S Lambdin B Gloyd S Integration of HIV/AIDS services into African primary health care: lessons learned for health system strengthening in Mozambique - a case study J Int AIDS Soc 13 3 10.1186/1758-2652-13-3 PMC2828398 20180975

[B9] Marchal B Cavalli A Kegels G Global health actors claim to support health system strengthening: is this reality or rhetoric? PLoS Med 2009 6 e1000059 10.1371/journal.pmed.1000059 19399158 PMC2667637

[B10] Instituto Nacional de Estatistica Censo Mocambique 2007 Maputo 2008

[B11] The Global Fund to Fight HIV/AIDS Tuberculosis and Malaria Data Quality Audit Tool http://www.theglobalfund.org/documents/me/Guidelines_LFA_DQ_Verifications.pdf

[B12] UNICEF http://www.unicef.org/statistics/index_24302.html

[B13] ORC Macro http://www.measuredhs.com

[B14] Forster M Bailey C Brinkhof MW Graber C Boulle A Spohr M Balestre E May M Keiser O Jahn A Egger M Electronic medical record systems, data quality and loss to follow-up: survey of antiretroviral therapy programmes in resource-limited settings Bull World Health Organ 2008 86 939 947 10.2471/BLT.07.049908 19142294 PMC2649575

[B15] Doherty T Chopra M Nsibande D Mngoma D Improving the coverage of the PMTCT programme through a participatory quality improvement intervention in South Africa BMC Public Health 2009 9 406 10.1186/1471-2458-9-406 19891775 PMC2777166

[B16] Nash D Elul B Rabkin M Tun M Saito S Becker M Nuwagaba-Biribonwoha H Strategies for more effective monitoring and evaluation systems in HIV programmatic scale-up in resource-limited settings: Implications for health systems strengthening J Acquir Immune Defic Syndr 2009 52 Suppl 1 S58 62 19858942 10.1097/QAI.0b013e3181bbcc45

